# Supplementary material for: Are carbon emissions trading and green financial instruments synergistic? -Comprehensive quantitative research based on content analysis
Source: PLoS One. 2024 Mar 7;19(3):e0298601. doi: 10.1371/journal.pone.0298601 (PMC10919601; doi:10.1371/journal.pone.0298601)
Supplement: S1 Data — (ZIP) [file pone.0298601.s001.zip › Data/地级市Z_F工作报告.zip!/╜¡╬≈/╔╧╚─╩╨/└┤╫╘╣1⁄2╓┌║┼í╛┬φ┐╦╩2╛▌═°í┐_2018.docx]

各位代表：

　　现在，我代表市人民政府向大会报告工作，请予审议，并请各位政协委员和列席会议的同志提出意见。

　　一、2017年工作回顾

　　刚刚过去的2017年，是党的十九大胜利召开之年，也是全面贯彻落实省第十四次党代会、市第四次党代会精神的第一年。在省委、省政府和市委的坚强领导下，在市人大、市政协的监督和支持下，全市上下紧紧围绕“决胜全面小康、打造大美上饶”总目标，深入贯彻新发展理念，坚持发展为要、项目为先、民生为本，统筹做好稳增长、促改革、调结构、优生态、惠民生、防风险等各项工作，全面完成了市四届人大二次会议确定的目标任务。全市财政总收入318.7亿元，增长5.5%，税占比较上年提高5.9个百分点。预计生产总值突破2000亿元、达到2070亿元，增长9%；固定资产投资突破2000亿元、达到2010亿元，增长12.3%；规模以上工业增加值增长9%；社会消费品零售总额815.6亿元，增长13.1%；银行业金融机构存款余额突破3000亿元、达到3250亿元，增长19.7%；贷款余额突破2000亿元、达到2210亿元，增长26.9%；城镇居民人均可支配收入突破3万元、达到31770元，增长9%；农村居民人均可支配收入12150元，增长9.5%。一年来的主要工作和成效是：

　　（一）项目攻坚势头强劲。始终把项目建设作为经济增长的主引擎、转型升级的主抓手，积极扩大有效投资，全面激发投资活力。全市23个省重点建设项目完成投资156.8亿元，142个省大中型项目完成投资565.3亿元，148个省市县三级联动集中开工推进项目完成投资406.4亿元，分别完成年度计划的125%、116%、112%。民间投资活力持续释放，预计完成1450亿元，增长10.1%。健全和落实市领导挂点协调、项目进展微信通报和总巡查、总督查、总协调等一系列推进机制，形成了“全方位谋划项目、全天候调度项目、全过程服务项目”的浓厚氛围。上饶铁路西货站、上饶动车组存车场、上饶国际综合物流园、信江高等级航道等一批重大基础设施项目开工建设。三清山机场建成通航，开辟了上饶走向全国的“空中通道”！九景衢铁路建成运行，全省人口第一大县鄱阳告别了不通火车的历史！

　　（二）产业发展提质增效。深入实施工业“五年决战七千亿”战略，预计全年完成工业投资1100亿元，占全市固定资产投资的55%；完成工业主营业务收入3600亿元，增长15%；工业对财政的贡献率25.1%，较上年提高3.8个百分点。“两光一车”产业快速发展，晶科能源组件出货量、光电转换率稳居全球第一，上饶光学镜片销量占据全国近三分之二市场，汉腾汽车开向全国所有省份，开出国门开进俄罗斯、伊朗。上饶正朝着“世界光伏城”“中国光学城”“江西汽车城”阔步前进！深入推进质量兴农、绿色兴农，围绕“东柚、西蟹、南红、北绿、中菜”产业布局，加快农业产业结构调整步伐，加速推进现代农业示范园建设，累计新增特色产业面积65万亩，全市66个特色农业园核心区面积达17.1万亩。上饶正朝着农业强市奋力前进！深入开展国家全域旅游示范区创建，铅山武夷山列入世界文化与自然“双遗产”名录，弋阳龟峰获评5A级景区，新增4A级景区3家，全市4A级及以上景区数量达到28个；率全省之先实现“县县有4A”，成为全国拥有4A级及以上景区最多设区市之一。全市接待游客1.6亿人次，增长30.3%；旅游总收入1480.9亿元，增长31.5%。上饶正朝着全域旅游新时代奋勇前进！

　　（三）经济新动能加快培育。大数据产业率先发展，上饶成为全省唯一的江西数字经济示范区。高铁经济试验区获批全省首个“大数据产业基地”，大数据产业园成为全省首个数字经济小镇；华为云数据中心基本建成，江西省政务数据灾备中心、知识产权联合交易中心等项目相继落户；数字文化新业态加快发展，千亩游戏产业园项目全面启动，引进韩国娱美德、上海恺英网络等文娱企业16家，贪玩公司实现主营业务收入20亿元、纳税2250万元；大数据呼叫中心高点起步，成功引进滴滴出行等业内顶尖的呼叫外包企业。上饶已成为全省数字经济发展“排头兵”。大健康产业迅速发展，成功入选全国首批“国家中医药健康旅游示范区”创建单位，海普洛斯基因组中心已经挂牌，20万人份江西多能干细胞库项目基本建成，腾讯医学人工智能项目成功落户。科技创新步伐加快，净增高新技术企业76家，新增院士工作站4个、博士后创新实践基地4家、国家级“星创天地”3家，全社会研究与试验发展经费支出占GDP比重稳步提高。

　　（四）城乡品质明显提升。“大美上饶”的城市底色更加亮丽！新型城镇化水平稳步提高，全市城镇化率突破50%。中心城区实施城建项目131个，完成投资106.8亿元。城市交通更通达，上饶大道北段、天佑大道西段、稼轩大道、吴楚大道、明叔大道实现通车，成功入选“十三五”国家公共交通都市创建城市。城市功能更完善，“去杂乱、补短板”工程扎实推进，新建综合管廊6.9公里、城市公厕21座，新建小公园、小游园3个，新增停车泊位2916个，建成城市绿道30公里，拆除违章建筑125.9万平方米。城市棚改更有力，累计完成拆迁1200万平方米，占全省总量的三分之一以上，信州区平均一天拆迁1万平方米。“大美上饶”的农村“颜值”令人赞叹！秀美乡村建设更出彩，完成村点建设3933个，整治提升通道58条，实施房屋立面“徽派”改造近12万栋。农村道路更通畅，完成国省道改造313公里、农村公路建设1800余公里、危桥改造94座。电子商务进乡村更便利，新建农村电商县级运营中心17个、乡镇电商服务中心63个，预计完成全社会电子商务交易额660亿元，稳居全省第二。

　　（五）深化改革纵深发力。扎实推进“放管服”改革，出台《关于推进“最多跑一次”改革方案》，精简各类行政权力89项，梳理公布第一批“一次不跑”事项196项。深入抓好“三去一降一补”专项行动，全年关闭退出煤矿20处，完成“去产能”101万吨，减轻企业负担超过95亿元，工业企业每百元主营业务收入成本下降1.2元。深化投融资体制改革，推动企业直接融资，新增“新三板”挂牌企业2家、进入上市辅导期企业2家、在江西联合股权交易中心挂牌企业675家；利用“贫困县IPO绿色通道”政策，引进拟挂牌上市企业71家，列全省第一。深化国资国企改革，促进国有企业做大做强，预计全市国有企业资产总额突破2000亿元、净资产突破1000亿元。深化城市管理和执法体制改革，城市管理和行政执法局挂牌成立。积极推进户籍制度改革，完成户口一元化改登61.2万人。深化商事制度改革，全面实施“多证合一、一照一码”登记制度，创新创业活力迸发，全市新增纳税人登记户21378户，增长34.2%。扎实推进农村宅基地管理试点工作，拆除房屋33.9万平方米，退出土地35.2万平方米。农村土地确权任务全面完成，顺利通过省级验收。

　　（六）扩大开放成效显著。持续加大招商引资力度，组织参加省（部）级招商活动13次。成功举办全球饶商回归大会、投资上饶绿色产业（北京）恳谈会、“两光一车”产业项目推介会。全年签约招商项目367个、总金额2223.4亿元，其中工业项目314个、总金额1340.7亿元。深入推行“抓洽谈签约、抓开工竣工、抓核查巡查、抓分析调度”工作机制，新签约项目注册率、开工率分别达到90.7%和80.4%。全年新批外商投资企业24家，实际进资9.4亿美元；新增“走出去”企业5家，直接对外投资311万美元；外贸进出口48.7亿美元，增长35.3%，总量、增幅位居全省前三。上饶“无水港”发运集装箱3.9万标箱，上饶—宁波“海铁联运天天班”开行305趟。

　　（七）生态文明建设扎实推进。国家生态文明试验区建设全面启动，生态文明建设“七大行动”“六大工程”扎实推进。绿色生态优势进一步巩固提升，完成植树造林16.1万亩，抚育森林66.5万亩，全市森林覆盖率达到61.9%。大力发展循环经济，推动形成绿色生产方式，万元GDP能耗预计下降4%。深入开展“净水、净空、净土”行动，主要河流断面水质达标率95%，城市空气质量优良率88.2%，实施水土流失综合治理33.2万亩，土壤环境质量得到改善；基本划定生态空间保护红线7238.5平方公里，占全市国土面积的31.8%。上饶荣获“国家森林城市”称号。

　　（八）民生事业持续改善。精准脱贫攻坚战全面打响，完成建档立卡贫困户搬迁5296人，实施产业扶贫项目1015个，预计219个省级贫困村、13.1万贫困人口脱贫，贫困发生率由4.95%下降到3.02%。尤其是光伏扶贫照亮了贫困群众脱贫致富路，全市建成村级电站97个，惠及贫困户5.2万户。上饶县、横峰县作为2017年脱贫摘帽县将接受国检。千方百计加大民生投入，全市财政民生八项支出388.9亿元，占一般公共预算支出的70.8%。省里确定的50件民生实事全部办成，中心城区10件民生实事全面启动。落实积极的就业，全年新增城镇就业6.7万人，新增转移农村劳动力9.7万人。加快完善社会保障体系，征缴五大社保基金86.7亿元，争取上级转移或配套资金47.4亿元，在全省率先完成全民参保登记工作。城乡医保整合全面完成，顺利接入国家异地就医结算平台。优先发展教育事业。全市新（改、扩）建校舍85万平方米，改造薄弱学校421所，消除超大班额1803个，弋阳、万年、三清山通过国家义务教育均衡发展督导评估。上饶幼儿师范高等专科学校挂牌开学，华东师范大学“方志敏基础教育园区”全面开工，江西婺源茶业职业学院、江西汉氏生命科技学院纳入“十三五”期间省高等学校设置规划。第2届中国“互联网+教育”峰会成功召开，我市已成为全国率先部署“腾讯智慧校园”的设区市。“健康上饶”战略扎实推进，组建医联体25个，以取消药品加成为标志的县级以上公立医院改革实现全覆盖，彻底终结了“以药补医”的历史。疟疾防治工作达到国家消除标准。抓好新形势下计划生育工作，全市人口出生率13.4‰，出生政策符合率92%。大力发展文化体育事业。投入资金21亿元，建设文化场馆1417个。成功举办第十四届赣浙闽皖四省四市民间艺术节、首届上饶文化创意产业博览会以及上饶国际半程马拉松赛、玉山中式台球世锦赛、市第四届运动会等文体盛会。

　　（九）社会保持和谐稳定。法治上饶建设进程加快，“七五”普法全面启动；法律援助制度不断完善，人民调解工作取得新成绩，公证机构体制改革全面完成。全面落实安全生产“党政同责、一岗双责”，扎实开展安全生产大检查、“安全生产执法年”、夏季百日安全专项行动，坚决守住安全发展底线，全年未发生较大以上安全事故。“信访积案化解攻坚”活动深入开展，党的十九大期间实现进京非访“零登记”。食品药品安全有效保障，食品安全追溯体系建设初见成效。社会治理创新取得新进展，网格化管理进一步深化。应急管理得到加强，夺取了“6·24”抗洪救灾工作全面胜利。“平安上饶”建设扎实推进，公安机关“一案一码”执法管理新模式成为全省样板，“赣剑”行动网上逃犯清网率全省第一，全市刑事案件发案率下降17.1%，人民群众安全感进一步提升。婺源荣获全国社会治安综合治理最高奖“长安杯”。

　　此外，统计、审计、工会、妇女儿童、民族宗教、档案、保密、农垦、人民防空、国家安全、外事侨务、涉台事务、民兵预备役、国防动员、防震减灾、检验检疫、无线电管理、住房公积金、科学普及、社科研究、机关事务、地方志、气象、水文、邮政、通讯、石油、烟草、粮食、盐业、老龄、关心下一代、老年教育、红十字、残疾人、慈善等事业都取得新成绩。

　　过去一年，我们着力加强政府自身建设，优化政务环境，努力塑造良好的政府形象。深入学习贯彻党的十九大精神，积极推动“两学一做”学习教育常态化制度化，严格落实中央八项规定精神，认真履行“一岗双责”，坚持不懈抓好作风建设。严格控制机构编制，财政供养人员实现“只减不增”。加强机关效能建设和行政审批制度改革，不断提升政府公信力和执行力。自觉接受人大法律监督和政协民主监督，办理人大代表建议96件、政协提案217件，办复率均为100%。坚持依法治市，推进依法行政，加大行政监察、审计监督和政务公开力度，党风廉政建设和反腐败工作得到全面加强。

　　各位代表！过去一年取得的成绩，是省委、省政府和市委统揽全局、正确领导的结果，是市人大、市政协有效监督、鼎力支持的结果，是全市人民锐意进取、奋发有为的结果。在此，我代表市人民政府，向付出辛勤劳动的广大干部群众，向全市人大代表、政协委员、各民主党派、工商联、无党派、各人民团体和社会各界人士，向关心支持上饶建设的离退休老干部、老同志，向驻饶部队、武警消防官兵和公安干警，向在我市创业的投资者、建设者，向中央和省驻饶单位及所有关心、支持上饶改革发展的同志们、朋友们，致以崇高的敬意和衷心的感谢！

　　总结过去一年的工作，全市经济欠发达状况有所改观，但发展不平衡不充分的问题仍然突出。主要是：经济总量不大、发展质量不高、发展活力不足、综合竞争力偏弱；环境保护压力较大，公共服务资源不足、水平不高，民生领域短板仍然较多；少数干部的精神状态、工作作风、能力本领与新时代发展的需要还有不少差距，“中梗阻”现象在一些部门仍然不同程度的存在。对于这些问题，我们将高度重视，继续采取有力措施，在今后工作中认真加以解决。

　　二、2018年工作安排

　　今年是贯彻党的十九大精神的开局之年，是改革开放40周年，是决胜全面建成小康社会、实施“十三五”规划承上启下的关键一年。做好今年的政府工作，总体要求是：全面贯彻党的十九大、中央经济工作会议以及省委十四届五次全会精神，以习近平新时代中国特色社会主义思想为指导，深入实施省委“创新引领、绿色崛起、担当实干、兴赣富民”工作方针，按照高质量发展的要求，坚持稳中求进工作总基调，坚持新发展理念，坚持以供给侧结构性改革为主线，统筹推进稳增长、促改革、调结构、优生态、惠民生、防风险各项工作，促进经济社会持续健康发展，全面加快“决胜全面小康、打造大美上饶”历史进程，着力打造省域副中心城市和赣浙闽皖四省交界高铁枢纽城市，奋力开创新时代上饶改革发展新局面。

　　主要预期目标是：生产总值增长8.5%左右；财政总收入增长7%左右；规模以上工业增加值增长8.6%左右；固定资产投资增长11%左右；社会消费品零售总额增长12%左右；外贸出口增长3%左右；实际利用外资增长9%左右；城镇和农村居民人均可支配收入分别增长8.5%、9%左右；居民消费价格总水平涨幅控制在3%以内；城镇登记失业率控制在4%以内；节能减排完成省政府下达的任务。

　　按照上述目标要求，我们将重点抓好五个方面工作：

　　（一）聚焦发展质量提升，着力做优现代产业体系。把发展经济的着力点放在实体经济上，主攻方向放在提高供给体系质量上，不断深化供给侧结构性改革，着力构建现代化经济体系。

　　抓实体工业，加快打造三个千亿级产业。持续深入实施“五年决战七千亿”战略，咬定“世界光伏城”“中国光学城”“江西汽车城”目标，支持金属新材料企业加大改造升级力度，打造“两光一车”升级版，加快形成金属新材料、光伏、汽车等三个千亿级产业，持续巩固上饶“全国三大光学产业基地”地位，推动上饶工业涅槃重生，脱胎换骨。继续实施晶科能源“双倍增”计划。加快建设中电海康智慧物联网产业园、华芯科技芯片封装、维真裸眼3D等项目。开工建设长安跨越新能源汽车项目，推动汉腾二期、中汽瑞华、爱驰亿维、博能商用车等新能源汽车项目年内建成投产。认真落实《信江河谷城镇群和滨湖板块工业发展规划》，开展“县域工业发展攻坚年”活动。支持各县（市、区）围绕既定的3个以内主导产业尤其是首位产业，大干快上、竞相发展，打造各具特色的工业板块，确保全市绝大部分县（市、区）园区主营业务收入过200亿元，力争上饶经开区主营业务收入突破1100亿元。学习、放大上饶经开区效应，鼓励有条件的县（市、区）与上饶经开区合作建园，错位发展，打造“飞地经济”。支持信州区在新320国道开辟工业新战场，支持铅山县建设上铅快速通道工业基地。坚持和完善“领导挂点联系企业”“市县主要领导每月工业日”等工作机制，进一步强化达规工业企业“入统”工作，全市新增规模以上工业企业250家，规上工业主营业务收入突破4000亿元。

　　抓现代服务业，加快推进大数据、大健康、大旅游产业升级。按照“江西省数字经济示范区”的定位，加快发展大数据、游戏、呼叫等新经济业态，加快推进中科院数创园、国美、网库、艾漫数据等项目，着力打造国家级的数字经济小镇。支持中科院云计算中心大数据研究院做大做强，开工建设大数据创新创业中心，确保大数据文创中心建成运营。抢先发展数字文化新业态，加快建成游戏产业园，积极打造游戏产业交易平台，推动恺英网络、韩国娱美德、贪玩等游戏企业做大做强，力争用两年时间形成百亿级产业，努力打造江西最大的游戏产业城。加快推进滴滴科技城项目建设，大力引进呼叫和服务外包企业，力争三年内呼叫席位突破3万个，努力打造江西最大的呼叫产业城。按照“生命健康城”的定位，加快建设上饶国际医疗旅游先行区，积极创建国家中医药健康旅游示范区，继续抓好干细胞、生殖健康、基因检测等项目，大力引进中医药加工、高端医疗、养生养老等项目。按照“中国东部旅游目的地、中转地、集散地”的定位，推进上饶市《“十三五”旅游规划》《全域旅游规划》《乡村旅游规划》“三规合一”，支持篁岭、鄱阳湖国家湿地公园创建5A级景区，加快推进高铁农都、水上乐园、城东文化旅游综合体、城西“十里槠溪”、上饶野生动物世界、影视文化小镇、国际医疗健康小镇、国际旅游港、云谷田园综合体、三宝街等“十大旅游综合体”项目。抓好商贸流通业，鼓励商业综合体和城区商圈错位发展、互补共赢。深入推进电子商务进农村、进社区、进企业，大力发展跨境电商，支持晶科能源实现跨境交易额超百亿。引导金融机构加大对实体经济的支持力度，大力发展绿色金融，支持融资租赁业发展，争取政策性、开发性金融机构稳定贷款投放，构建良好的金融环境。

　　抓现代农业，加快建设农业强市。坚持质量兴农、绿色兴农，继续实施“十百千万”工程；围绕“东柚、西蟹、南红、北绿、中菜”产业布局，编制和落实特色产业发展规划。瞄准市场需求发展绿色农业、特色农业、品牌农业和中药材产业，大力推进优质稻、蔬菜、果业、茶叶、水产、草食畜、中药材、油茶、笋竹、休闲农业和乡村旅游“十大工程”。着力推进农村土地有序流转，通过土地股份合作、土地托管、代耕代种等多种方式，引导土地经营权向农业龙头企业、农民合作社、家庭农场流转。落实粮食安全省长责任制，建成高标准农田55万亩。促进农村一二三产业融合发展，支持德兴发挥“中医药+”优势，做大中医药健康养生产业；支持铅山农产品精深加工中心、横峰食品加工产业园、鄱余万食品加工及稻虾（蟹）共作示范基地建设，提升农产品加工业发展水平。加大万年贡米、婺源绿茶、广丰马家柚、德兴红花油茶等名优农特产品的整合、包装、宣传、推介力度，进一步打响上饶农产品品牌。

　　抓创新驱动，加快培育发展新动能。进一步强化创新引领作用，继续加大全社会研发投入，支持中科创新研究院设立科技创新投资引导基金，扎实开展“科贷通”、知识产权质押融资试点，撬动企业扩大科技创新资金投入。积极培育高新技术产业基地、星创天地、众创空间、科技企业孵化器等创新平台，全年新增国家高新技术企业30家、省级高新技术产业园1家，新组建省级工程技术研究中心3个，申请专利5500件。深入实施“信江英才866”、“饶城英才计划”等引才工程，积极引进外国专家和海外工程师，不断壮大科技人才队伍。把“饶商回归”和“饶才回归”结合起来，在全社会大力弘扬企业家精神和工匠精神；鼓励重点企业争创国家级质量品牌，加强高技能人才培训基地和技能大师工作室建设，大力培育“上饶工匠”。支持上饶师院、江西医专、上饶幼专加大人才引进、项目建设、学科发展的力度，为全市经济社会发展提供人才和智力支持。

　　（二）聚焦发展格局优化，着力提升城乡品质。大力实施区域协调发展战略，增强城乡发展的整体性、协调性。

　　抓重大项目带动，优化提升区域发展格局。着眼于把上饶打造成全国性综合交通枢纽，优先构建现代综合交通运输体系。积极配合昌景黄高铁项目建设。大力支持通用机场及直升机起降点建设。加快推进鄱阳至余干高速公路、老320国道上饶段全线升级改造、上饶铁路西货站、上饶动车组存车场等项目建设。力争开工建设万年至鹰潭北一级公路。继续做好上饶城市轨道交通线、旅游轨道交通线、上饶至浦城高速公路等项目前期工作，启动德兴至景德镇高速公路项目前期工作。切实抓好重大电力项目建设，继续推进上饶发电厂项目，加快实施园北、白云220千伏输变电工程，建成紫阳110千伏输变电工程，确保上信500千伏线路建成投运。优化城镇体系格局，统筹推进中心城区、县城和重点镇建设，完成水南片区、国际医疗旅游先行区、老城区控规编制及城市设计；加快推进信江河谷城镇群建设，打造一体化的城镇群综合体，着力构建城乡互促共进、区域协调发展的新型城镇体系。补齐鄱余万滨湖地区发展“短板”，深入实施《支持鄱余万三县基础设施建设和民生事业发展行动方案》，突出抓好项目对接和推进，加快鄱余万小康攻坚步伐。

　　抓中心城区首位度提升，充分发挥龙头辐射带动作用。积极推进上饶县撤县设区，推动广丰全面融入中心城区，实现“一城三区”组团式发展。全面开展生态修复、城市修补工作，启动实施一批城市“双修”项目，综合治理“城市病”。继续抓好城市棚户区改造，力争用1—2年时间，再完成各类棚户区改造5万户，拆迁面积1000万平方米。深入实施“去杂乱、补短板”工程，继续抓好城市路网、学校、医院、小游园、文化场馆、公共服务中心、停车场、菜市场、专业集贸市场、公交站台等项目建设，进一步完善城市功能，提升城市品位。扎实开展“城市管理提质年”活动，强化社区物业管理，开展老旧小区整治提升，让生活环境更宜居。

　　坚持“言必信、行必果”，接续推进近年来谋划的一批城建项目和去年启动实施的10件民生实事落地见效。道路贯通方面，确保天佑大道、茶圣东路、吴楚大道、滨江东路、稼轩大道北延伸等5条城区主干道建成通车；全面完成大茅山路等高铁新区10条道路建设，加快推进仕诠路、元杰路、槠溪南路等20条城区次干道（支路）贯通项目；开工建设云碧峰大桥、丁家洲大桥。功能完善方面，加快推进市人民医院城北院区、老年人综合活动中心、市档案馆、市美术馆等项目；开工建设市委党校新校区、市一中新校区、市三中、信美学校、叶挺学校、带湖学校、明叔小学、市十五小、上饶幼专附属幼儿园；开工建设市妇女儿童中心、青少年活动中心、演艺中心、社会职工之家；开工建设四脚亭集贸市场、城东集贸市场、综合客运枢纽蓄车停车场及城南、城北公交综合场（站）。形象提升方面，加快推进凤凰大道、紫阳南大道、五三大道改造提升；启动书院路、丰溪路、庆丰路、茶圣路、玉丰路和广丰路口改造提升；开工建设信江两岸灯光秀及夜游项目，统筹推进金龙岗、普济巷等10条里弄小巷改造。

　　抓乡村振兴战略实施，促进城乡融合发展。推动城乡要素融合，加快推进城市资本、技术、人才下乡，实现城乡要素双向融合互动和资源优化配置。按照“产业兴旺、生态宜居、乡风文明、治理有效、生活富裕”的总要求，继续高标准推进3700个秀美乡村点建设，把农村建成生态宜居的美丽家园。提升农村建房整体风貌，将融入赣东北民居建筑特色的“徽改”进行到底。加大农民文化教育和技能培训力度，积极培育爱农业、懂技术、善经营的新型职业农民。加强乡风文明建设，倡导“婚事新办、喜事小办、丧事俭办”文明新风尚，推动农村移风易俗，让文明乡村既有“颜值”又有气质。健全自治、法治、德治相结合的乡村治理机制，建立健全村务监督委员会，充分发挥村民理事会、村“五老”等各类主体的积极作用，引导村民共同办好自己的事情。完善公共资源优先向农业农村倾斜的政策体系，推动城乡水电路气网等基础设施互联，加快公共服务向农村覆盖。大力实施大中型灌区续建配套、病险水库除险加固、农村饮水安全工程等水利项目，建设农村公路1000公里，新（改）建桥梁80座。

　　（三）聚焦发展动力增强，着力深化改革开放。坚持以问题为导向加大改革攻坚力度，持之以恒提升开放型经济水平，进一步激发市场活力，增强发展动力。

　　抓改革攻坚强动力。深化“放管服”改革，继续取消或下放一批行政权力，全面推开“最多跑一次”改革，扎实推进实体政务大厅及政务服务标准化建设，推进“互联网+政务服务”，逐步实现“一次不跑”。持续深入开展“降成本、优环境”专项行动。抓好国地税征管体制改革，优化国地税联合办税服务。深化国有企业市场化改革，积极推动市属国资国企集中统一监管，支持市属国有企业整合重组，建立完善授权监管方式，着力提升国有企业多元化经营水平，增强企业盈利能力。按照省政府统一部署，稳步推进事业单位公车改革。深化医药卫生体制改革，完善“医保、医疗、医药”三医联动机制，积极开展分级诊疗、医联体、家庭医生签约服务、药品购销“两票制”等工作，逐步建立现代医院管理制度和薪酬制度。全面完成农村集体建设用地、房地一体的农村宅基地、农垦国有土地使用权确权登记颁证工作，建设县级农村综合产权流转交易市场。深化农垦改革，推进垦区集团化、农场企业化。稳步推进农村集体产权制度改革，发展壮大农村集体经济，下大气力消灭集体经济“空壳村”。全面加强农村宅基地管理，严格落实“一户一宅”，不断提高农村建房用地集约化水平。

　　抓开放升级增活力。开展主导产业龙头项目“招大引强”竞赛活动，全市围绕“两光一车”产业，各县（市、区）围绕本地主导产业尤其是首位产业，重点面向世界500强、中国500强和行业20强企业，坚持“一把手”激情招商，实行产业引导基金招商，引进一批达产后主营业务收入超30亿元、能够支撑发展全局的重大项目。充分利用“香港周”“赣台会”“绿发会”等招商平台，每季度举办一次专题招商活动。继续强化“四抓”工作机制，倒逼招商项目早落地、早建设、早投产、早“入统”，切实提高招商引资实效。优化对外贸易结构，加大生产型出口企业引进和培育力度。加快上饶国际综合物流园建设，确保物流大道年内建成通车。积极推进“海铁联运天天班”双向对开，力争开通上饶至哈萨克斯坦、白俄罗斯、俄罗斯的中欧（亚）国际货运班列。全年引进省外资金、利用外资、外贸出口分别达到700亿元、12.2亿美元、336.8亿元。

　　抓发展平台提升承载力。坚定支持上饶经开区继续做大做强，推动上饶高新区创建国家级高新区，打造工业“双引擎”。加大标准厂房建设力度，确保上饶经开区建成标准厂房150万平米以上，实现企业“拎包入驻”。强化集约节约用地，严把供地关、严查履约率，鼓励建设多层厂房，向空中要地，开展闲置土地清理，确保新增工业项目平均投入强度每亩达到300万元以上，提升园区“两率一度”水平。加快工业云、智慧园区信息化服务平台建设，培育一批省、市两化融合示范企业和示范区。加快推进上饶高铁经济试验区、国际医疗旅游先行区核心区基础设施和项目建设，确保江天农产品冷链物流园一期项目竣工开业、二期开工建设，抓好江西汉氏联合医院、江西汉氏生命科技学院、上饶国际精准医疗中心等项目建设。大力推进现代农业示范园建设，加大农业招商力度，不断做强核心区、提升示范区、扩大辐射区。启动实施《上饶市促进军民融合发展行动计划》，积极创建一批军民融合产业示范区、示范基地，支持德兴航空产业基地建设，促进军民融合发展。

　　（四）聚焦发展方式转变，着力巩固生态优势。牢固树立社会主义生态文明观，加大生态环境保护力度，加快发展绿色生态产业，推动形成人与自然和谐发展现代化建设新格局。

　　抓生态环境治理，坚决打好污染防治攻坚战。坚持全民共治、源头防治，着力解决突出环境问题。实施“净空”行动，切实抓好工业园区污染治理、施工扬尘、油烟、尾气整治等污染防控，坚决打赢蓝天保卫战。实施“净水”行动，大力提升中心城区污水管网贯通率，完成三江片区污水管网建设，全面消灭全市劣V类水，大力整治农村面源污染，守护好鄱阳湖的“一湖清水”。实施“净土”行动，全面推进生活垃圾处理设施建设，原则上每个县（市、区）建设1个生活垃圾焚烧发电厂。继续深入开展城乡环境综合整治，集中治理砖瓦窑，整治农村乱埋乱葬，切实解决城乡环境“顽疾”。抓城市公厕改造，促景区厕所提升，补农村改厕短板，深入推进“厕所革命”。采取政府购买服务的方式，全面推开农村垃圾处理市场化运营。积极引导城乡居民自觉开展垃圾分类，不断提高垃圾处理减量化、资源化、无害化水平。

　　抓绿色经济培育，推动绿色低碳循环发展。加快资源利用方式转变，抓好有色金属等传统产业绿色化改造，加快推进广丰卷烟厂易地技改、万年水泥厂环保搬迁等项目。积极稳妥推进“十三五”期末全市煤炭行业整体退出、烟花爆竹行业基本退出，完成退出煤矿产能90万吨。大力发展生态农业、中药产业、林业经济和林下经济，积极发展生态旅游、健康养老、休闲度假等服务业，着力构建绿色产业体系，推动形成绿色发展方式。倡导简约适度、绿色低碳的生活方式，重视发展清洁能源，积极推进“电能替代”。反对奢侈浪费和不合理消费，开展创建节约型机关、绿色家庭、绿色学校、绿色社区和绿色出行等行动。加快健全绿色金融服务体系，建立企业环境信用评价系统，引导实体经济加快绿色转型发展。

　　抓生态系统保护，加快完善生态文明体制机制。建立健全空间管控体系，全面推行“多规合一”，严格生态保护红线、永久基本农田、城镇开发边界管控。积极探索山水林田湖草综合治理模式，加强地质灾害防治，提升自然生态系统的稳定性。进一步推进国土绿化，大力实施长江防护林、退耕还林等生态修复工程，完成植树造林13.3万亩、退化林修复18.6万亩。完善自然资源开发保护、生态补偿等制度。积极推进自然资源统一确权登记工作，健全和落实控制污染物排放许可制度。推动“环保警察”队伍建设。完善提升“河长制”升级版。积极开展第二次全国污染源普查和省以下环境监测监察机构垂直管理改革工作。

　　（五）聚焦发展成果共享，着力增进民生福祉。坚持以人民为中心的发展思想，加大保障和改善民生力度，既尽力而为，又量力而行，不断满足人民日益增长的美好生活需要。

　　扎实打好脱贫攻坚战。按照“脱真贫、真脱贫”的总要求，围绕稳定实现农村贫困人口“两不愁、三保障”目标，既不降低标准，也不吊高胃口，紧扣“精准”二字，下足“绣花”功夫，加快推进“产业扶贫全覆盖、健康扶贫再提升、易地搬迁再精准、教育扶贫再对接、贫困村庄整治再推进”五大工程，确保2018年1个贫困县、172个贫困村退出，9.96万贫困人口脱贫。聚焦重点区域，围绕全市重点贫困村和深度贫困村，推动脱贫新增资金、项目、举措予以重点倾斜。聚焦重点人群，强化对老年人、残疾人、重病患者等特殊贫困人口的精准帮扶。采取有针对性的帮扶措施，抓好城市贫困人口帮扶。健全资金运作、督查考评和贫困人口动态管理等制度，做好脱贫后续保障。注重扶贫同扶志、扶勤、扶德、扶智相结合，激发贫困人口内生动力。

　　着力提高社会事业发展水平。把教育事业放在优先位置，继续加大教育投入力度，加快推进义务教育学校标准化建设，新（改、扩）建校舍100万平方米，其中新建公办幼儿园51所。有序推进中心城区学校网点布局规划落实，确保鄱阳、余干、横峰、铅山顺利通过国家义务教育均衡发展督导评估。支持江西医专建成特色鲜明的应用型本科医学院校。优化计划生育服务管理，构建鼓励按政策生育制度体系。加大医疗卫生人才培养、医疗服务信息化和医院项目建设力度，重点培养全科医生、儿科医生和中医人才，基本建成上饶市智慧医疗云平台，确保市妇幼保健院（儿童医院）、市第二人民医院等项目投入使用。进一步激活乡镇（街道）综合文化站服务功能，建设村（社区）综合文化服务中心2100个，设立村级宣传文化管理岗位。积极培育发展数字文化新业态，支持动漫、影视、视觉艺术、文化设备制造、版权产业发展，办好第二届上饶文化创意产业博览会，确保市博物馆建成开放。统筹推进全民健身场地建设，强化体育设施有效供给。办好上饶国际半程马拉松赛、全国体育舞蹈国际邀请赛、中式台球世锦赛、全国竞走大奖赛等体育赛事。

　　加快完善社会保障体系。落实更加积极的就业政策，完善城乡就业创业服务体系，加大职业技能培训力度，确保新增城镇就业5万人、转移农村劳动力7.2万人。按照“兜底线、织密网、建机制”的要求，大力推进社会保险精准扩面、精准征缴，确保我市养老保险、基本医疗保险、失业保险、工伤和生育保险参保人数分别达到373.1万人、708万人、30万人、46.5万人和26万人。扎实推进跨省异地就医直接结算工作，全面推行多元复合式医疗保险支付方式改革，认真做好长期护理保险试点工作。加大社会救助力度，落实优抚双拥安置政策，抓好市救灾物资储备库建设。全面放开养老服务市场，支持发展社区养老服务，提升养老服务质量，就近就便满足老年人养老服务需求。

　　切实加强和创新社会治理。完成第十届村（居）委会换届选举工作。加快社会治安防控体系建设，严厉打击各类违法犯罪行为，维护良好社会秩序。加大依法治访力度，加强预防和化解社会矛盾机制建设，及时回应和办理群众诉求事项，全力维护群众合法权益。强化安全生产企业主体责任和行业、基层监管责任，扎实开展隐患排查治理、专项督查检查、监管执法活动，坚决遏制重特大安全事故发生。深入开展食品药品领域违法违规行为专项整治，加大“餐桌污染”治理力度，积极创建省级食品安全示范城市。坚持安全与畅通并重，积极推动城市交通管理信息化、智能化、精细化，全面提升道路交通治理水平。提高全民国家安全意识，推进全民安全教育场地建设。加快建设公共法律服务平台，规范基层法律服务工作。继续推进社区网格化管理，进一步整合资源、配强力量、发动群众，着力构建“网络全覆盖、工作无缝隙、服务零距离、管理无漏洞”的社会治理新格局。

　　各位代表，打铁必须自身硬！全市各级政府要深入贯彻落实党的十九大精神，始终恪守勤政为民的天职，切实加强政府自身建设。一是旗帜鲜明讲政治。把对党绝对忠诚放在首位，牢固树立“四个意识”，坚定不移地用习近平新时代中国特色社会主义思想武装头脑，坚定不移地维护习近平总书记核心地位，坚定不移地维护党中央权威和集中统一领导。扎实推进“两学一做”学习教育常态化制度化，认真开展“不忘初心、牢记使命”主题教育。始终把抓落实作为政府工作第一任务，严格遵守政治纪律和政治规矩，不折不扣贯彻落实好党中央、国务院、省委、省政府和市委的各项决策部署。二是着力推进依法行政。把依法行政作为基本准则，坚持法无授权不可为，法定职责必须为，严格规范公正文明执法，做好行政复议和行政应诉工作。坚持依法履职，杜绝法外设权，加快推进服务流程再造，努力营造公平、法治、高效的营商环境。大力推行政务公开，主动接受人大的法律监督、政协的民主监督和社会各界的监督，把人民群众的期待融入政府的决策和工作之中，让权力在阳光下运行。认真办理人大代表议案建议和政协提案，认真听取各民主党派、工商联、无党派人士和人民团体的意见。三是始终坚持以人民为中心的发展思想。作为人民政府，所有工作都要体现人民意愿、维护人民利益、接受人民监督。始终把人民对美好生活的向往作为奋斗目标，下大气力解决好群众最关心最迫切的问题，在幼有所育、学有所教、劳有所得、病有所医、老有所养、住有所居、弱有所扶上持续取得新进展，努力使政府各项工作与群众意愿“合拍”，与百姓心声“共振”，不断提升人民群众的获得感、幸福感、安全感。紧紧扭住解决群众身边的不正之风问题，加强政府效能建设，加快干部作风转变，严格绩效管理考核，不断提高政府及部门工作人员办事效率和服务水平。四是切实增强担当精神和实干本领。打造大美上饶，迫切需要政府的大美作为。政府公职人员既要政治过硬，又要本领高强，大兴学习之风，大兴调查研究之风，向基层学习，拜人民为师，不断提高“八大本领”。发扬钉钉子的精神，以真抓的实劲、敢抓的狠劲、善抓的巧劲、常抓的韧劲，抓铁有痕、踏石留印抓落实。始终以“功成不必在我”的境界谋发展，以“功成必定有我”的担当精神抓落实，把责任扛在肩上，勇于挑最重的担子，敢于啃最硬的骨头，善于接最烫手的山芋，切实把各项工作抓紧抓实、抓出成效。进一步加强督查落实，加强协同攻坚，对重要工作、重点任务、重大项目，必须明确时间表、路线图和责任人，确保各项目标任务按时保质完成。五是不断加强清廉政府建设。严格遵守中央八项规定实施细则，大力弘扬“爱国、创造、清贫、奉献”的方志敏精神，持之以恒纠“四风”、改作风，全力维护风清气正的政治生态。全面落实党风廉政建设责任制，强化工程建设、政府采购、资金使用等重点领域监管，发挥监察、审计监督作用，坚持有腐必反、有贪必肃、有责必问，促进干部清正、政府清廉、政治清明。

　　各位代表，新时代肩负新使命，新思想指引新征程！让我们更加紧密地团结在以习近平同志为核心的党中央周围，在省委、省政府和市委的坚强领导下，团结拼搏，扎实苦干，尽锐出战，精准发力，为加快“决胜全面小康、打造大美上饶”历史进程，为打造省域副中心城市和赣浙闽皖四省交界高铁枢纽城市而不懈奋斗！
